# *Spirulina platensis* alleviates chronic inflammation with modulation of gut microbiota and intestinal permeability in rats fed a high‐fat diet

**DOI:** 10.1111/jcmm.15489

**Published:** 2020-07-07

**Authors:** Ting Yu, Yan Wang, Xiaosu Chen, Wenjie Xiong, Yurong Tang, Lin Lin

**Affiliations:** ^1^ Department of Gastroenterology The First Affiliated Hospital of Nanjing Medical University Nanjing China

**Keywords:** gut permeability, high‐fat diet, inflammation, microbiota, *Spirulina platensis*

## Abstract

Recent research suggested that taking a high‐fat diet (HFD) may lead to a gut microbiota imbalance and colon tissue damage. This would lead to increased intestinal permeability and consequent constant circulation of low‐grade inflammatory cytokines. *Spirulina platensis* can protect against HFD‐induced metabolic inflammation and can stimulate the growth of beneficial bacteria in in vitro stool cultures. However, it is unknown whether this beneficial effect acts on intestinal tissues. In this study, rats were fed a high‐fat diet fed with 3% *S platensis* for 14 weeks. We analysed endotoxin, the composition of the microbiota, inflammation and gut permeability. We found that *S platensis* decreased the bodyweight and visceral fat pads weight of the HFD‐fed rats. In addition, it lowered the levels of lipopolysaccharide and pro‐inflammatory cytokines in serum. Our results showed that *S platensis* could largely reduce the relative amount of *Proteobacteria* and the *Firmicutes/Bacteroidetes* ratio in faecal samples from HFD‐fed rats. *S platensis* significantly reduced intestinal inflammation, as shown by decreased expression of myeloid differentiation factor 88 (MyD88), toll‐like receptor 4 (TLR4), NF‐κB (p65) and inflammatory cytokines. *S platensis* also ameliorated the increased permeability and decreased expression of tight junction proteins in the intestinal mucosa, such as ZO‐1, Occludin and Claudin‐1. Therefore, in HFD‐induced gut dysbiosis rats, *S platensis* benefits health by inhibiting chronic inflammation and gut dysbiosis, and modulating gut permeability.

## 1 INTRODUCTION

There is a positive link between high‐fat induced obesity and low levels of chronic inflammation, which is associated with the heightened pro‐inflammatory cytokine expression that contributes to many complicated metabolic disorders, for example, diabetes, hypertension, cardiovascular diseases and several types cancer.^1^, ^2^ Additionally, the gut microbiota contributes to the development of obesity, and dysbiosis of the gut microbiota is important in the obesity‐associated chronic inflammatory state.^3^, ^4^, ^5^ On the one hand, as the diversity and abundance of beneficial microbiota decreased, a high‐fat diet (HFD) would also lead to the gut microbiota dysbiosis. On the other hand, an HFD has the potential to increase the faecal level of lipopolysaccharide (LPS), and cause endotoxaemia or systemic low‐grade inflammation, which can further damage peripheral tissues.^6^, ^7^

The intestinal barrier, or the intestinal mucosal barrier, serves as the main barrier to protect against antigens or toxins carried in food. It is a single intestinal epithelial cell layer that is lined with tight junction proteins that modulate intestinal permeability.^8^, ^9^ Gut‐derived bacterial LPS extracted from rats fed an HFD stimulated increased induction of toll‐like receptor 4 (TLR4)‐dependent activation of the myeloid differentiation factor 88 (MyD88) and downstream NF‐κB signalling pathway, resulting in reduced expression levels of tight junction protein and colitis; thus, enhancing intestinal permeability.^10^, ^11^ In these circumstances, the intestinal barrier function will be negatively affected and the potential for bacterial translocation will increase. Bacterial translocation opens a gateway to various types of toxins, such as LPS, which cause localized inflammation and stimulate systemic inflammation via rapid increases in cytokine levels.^12^ From this perspective, regulating the gut microbiota could contribute to ameliorating the systemic chronic inflammatory response induced by an HFD and reduce intestinal damage.

*Spirulina platensis* (*S platensis*) is part of the family of Oscillatoriaceae^13^ that occurs naturally in alkaline lakes.^14^ As a functional resource, *S platensis* contains various active ingredients, such as vitamins, minerals, phenolic acids, beta‐carotene, proteins and tocopherols. It also exhibits high levels of antioxidant and anti‐inflammatory activities,^15^ including essential amino acids.^16^ These nutritional benefits have led to the use of *S platensis* as food additive for animals (such as birds and fish) or as food supplements for humans. *S platensis* and its active ingredient C‐phycocyanin have beneficial immunomodulatory, anti‐inflammatory, nephroprotective, hepatoprotective, antidiabetic, neuroprotective, anti‐cancer, anti‐hypertensive and antigenotoxic functions.^17^, ^18^, ^19^ Recently, researchers have considered using *S platensis* as a prebiotic source because it can benefit the growth of *Akkermansia*, *Lactobacillus* and *Butyricimonas*, and suppress the growth of *Clostridium* and *Dorea *in vitro. *S platensis* possess a regulatory effect that modulates the gut microbiota.^20^, ^21^, ^22^ Thus, the beneficial effects of *S platensis* in reducing obesity‐associated chronic inflammatory state are associated with intestinal activities. However, the regulation of intestinal barrier function as well as the improvement in intestinal tissue damage under HFD by spirulina platensis has not yet been studied. In addition, whether the protective effect of *S platensis* on intestinal barrier function related to LPS‐activated TLR4/MyD88/NF‐κB signalling pathway is not known. Therefore, in the present study, we used *S platensis* investigated the mechanism of its effects on the gut microbiota and intestinal permeability in HFD‐fed rats.

## 2 MATERIALS AND METHODS

### 2.1 Animal samples, experimental design and sample collection

Male SD rats weighing between 250 and 270 g were kept in a controlled environment room where the temperature ranged between 22 and 28°C and the humidity was maintained at around 60%, with a simulated natural light cycle of 12‐hour daytime (8:00‐20:00 hours) and 12‐hour night The light time is between 8:00 and 20:00.

After one week of acclimatization, we randomly divided the rats into three groups, each group containing eight individuals. The groups comprised low‐fat diet‐fed rats (LFD, 10 kcal% fat D12450B, control group), HFD‐fed rats (45 kcal% fat D12451, FBSH Biopharmaceutical Co., Ltd) and rats fed an HFD with 3% *Spirulina platensis* (SP group).^23^, ^24^ 100% pure *S platensis* powder was obtained from Lianmai Biotech Ltd. and administered it 3 g/100 g of diet to the experimental animals. We closely recorded each rat's bodyweight and food intake on a weekly basis. After feeding rats for 14 weeks, we collected faeces released by individual rats and stored them in a sterile tub. After 12‐hour fasting overnight, we collected blood samples from sacrificed rats and separated their serum using centrifugation (1000*g*, 10 minutes). Colon tissue was then excised, their faecal contents were discharged, and the tissue was rinsed using phosphate‐buffered saline (PBS). The colon samples were then stored in liquid nitrogen at −80°C. The Institutional Animal Care and Use Committee of Nanjing Medical University (Approval no. 201621533), Nanjing, China provided prior approval of our care of the animals and the experimental protocols used.

### 2.2 Biochemical analysis

We used a Fully Automatic Biochemistry Analyzer (Sigma‐Aldrich) to assay serum triglyceride (TG), total cholesterol (TC), low‐density lipoprotein cholesterol (LDL‐C), high‐density lipoprotein cholesterol (HDL‐C) and free fatty acid (FFA) in the samples. Commercial enzyme‐linked immunosorbent assay (ELISA) kits were used according to the manufacturer's protocols (Jiancheng) to analyse the interleukin (IL)‐6, tumour necrosis factor alpha (TNF‐α) and IL‐1β levels in serum.

### 2.3 Histological analysis

Colon tissues were rinsed with cold PBS, embedded in paraffin blocks, sectioned, mounted on slides, and stained with haematoxylin and eosin following established histopathology procedures. As described previously, we used a histological scoring system to assess inflammatory cell infiltration and tissue damage.^25^

### 2.4 Determination of oxidative stress‐related parameters

A commercial kit was used to assay reactive oxygen species (ROS) levels in colon tissues (STA‐347, Cell Biolabs Inc) according to the manufacturer's instructions and as described previously.^26^ Insoluble particles were removed from tissue lysates by centrifugation at 10 000 *g* for 5 minutes. The oxidative reaction in 50 μL of the supernatant was accelerated by adding 50 μL of the Catalyst (from the kit) and incubating for 5 minutes at room temperature. Then, 100 μL of 2′,7′‐Dichlorofluorescin Diacetate (DCFH)‐DiOxyQ probe solution was mixed with the samples to determine the total free radical levels (both ROS and reactive nitrogen species (RNS)). After incubation for 30 minutes at room temperature, the optical density of the samples was read using a fluorescence plate reader at Ex/Em = 480/530 nm. Malondialdehyde (MDA) levels were determined using a commercial kit from Sigma‐Aldrich (MAK085A). The MDA content was determined by the reaction of MDA with thiobarbituric acid (TBA) to form a fluorometric product (Ex/Em = 532/553 nm), proportional to the amount of MDA present. A commercial kit from Cell Biolabs Inc (STA‐340) was used to estimate superoxide dismutase (SOD) levels, according to the manufacturer's instructions and as described previously.^27^ SOD activity was determined by assessing the degree of inhibition of xanthine‐derived superoxide radical generation using xanthine oxidase, which forms a red formazan dye upon reaction with 2‐(4‐iodophenyl)‐3‐(4‐nitrophenol)‐5‐phenyltetrazolium chloride.

### 2.5 Faecal DNA extraction and gut microbiota analysis

From each group, we randomly selected four colon content samples, extracted their DNA and sequenced the 16S rRNA genes. Briefly, a Qiagen DNeasy Blood & Tissue kit (Qiagen) was used to extract total bacterial DNA in the colonic content, following the manufacturer's instructions. A Nanodrop 1000 spectrophotometer (Thermo Fisher Scientific) was used to determine the genomic DNA concentration, and electrophoresis on agarose gels were used to determine the DNA integrity.

For all collected samples collected, we standardized the genomic DNA concentration to 20 ng/μL. DNA extracted from two media was mixed in an equal volume to conduct further analysis. Relative bacterial abundance was assessed using 16S rRNA gene sequencing, which was performed at the laboratory of the Huada Gene Institute (BGI, Beijing, China). The hypervariable V4 region (515‐806) of the 16S rRNA gene was amplified using universal primers 515F (5′‐GTGCCAGCMGCCGCGGTAA‐3′) and 806R (5′‐GGACTACHVGGGTWTCTAAT‐3′). The detailed procedures were carried out as described previously.^28^ The raw research data were deposited at the NCBI sequence read archive database with accession number: SUB5962285. The details of the bioinformatic analyses described in a previous publication.^28^ An in‐house pipeline (GENOME) was used to obtain the high‐quality sequencing reads. A 97% identity cut‐off was used to cluster the operational taxonomic units (OTUs). We removed OTUs with a relative abundance < 0.005% to decrease the influence of low abundance, spurious OTUs. OTU principal component analysis (PCA) was used to construct a Venn diagram, and R software (v3.1.1) was used to produce the OTU rank curve. QIIME software (version, 1.7.0) and the OTU data were used for alpha diversity analysis (eg observed species, Shannon, Chao, Simpson, and Ace indices) and beta diversity analysis (diversity distance, unweighted UniFrac, and unweighted UniFrac cluster tree). To select potential microbial biomarkers, statistical analysis using SPSS 17.0 software (IBM Corp.; *P* < .05) was combined with LEfSe (linear discriminant analysis effect size, Linear discriminant analysis (LDA) score > 2.00). For the potential microbial biomarkers, pie charts (phylum level) and histograms (genus level) were created using GraphPad Prism 5 GraphPad Software, Inc).

### 2.6 Serum and faecal LPS

A quantitative chromogenic limulus amoebocyte lysate (LAL) QCL‐1000 test kit (LONZA) as used to measure serum and faecal LPS levels, following the manufacturer's instructions. Faecal and serum samples were prepared in the kit‐provided pyrogen‐free water. Heating at 75°C for 10 minutes in a water bath was used to deactivate the diluted serum samples. The samples were then incubated at 37°C for 10 minutes in 50 μL of LAL reagent, followed by addition of the of LAL chromogenic substrate (100 μL) and incubation for 6 minutes. The addition of 100 mg/mL of SDS was used to terminate the reaction, and the spectrophotometric detection at 405 nm was used to assess the yellow colour produced by substrate cleavage.

### 2.7 Gut permeability in vivo

In vivo permeability measurement was modified from previously described methods based on gut permeability to 4‐kD FITC‐dextran.^29^ Rats were fasted for 6 hours and gavaged with 4‐kD FITC‐dextran (360 mg/kg bodyweight, 10 mL/kg). After 1 hour, 200 μL blood was collected from the tip of the tail vein. Plasma was diluted in an equal volume of PBS (pH 7.4), and the FITC‐dextran concentration was determined with a Synergy 2 microplate reader (BioTek), with serial dilutions of FITC‐dextran used as a standard curve.

### 2.8 Immunofluorescence staining

Frozen sections of rat distal colon or cultured smooth muscle cells (SMCs) were washed using PBS, fixed at −4°C using 100% acetone, blocked using BSA for 60 minutes and incubated with primary antibodies recognizing TLR4 or MyD88 (Cell Signaling Technology). Binding of the primary antibodies was detected using rhodamine or fluorescein isothiocyanate (FITC)‐conjugated secondary antibodies (Bioworld). DAPI (2‐(4‐amidinophenyl)‐1H‐indole‐6‐carboxamidine; Sanofi‐Aventis, SA) was used to visualize the nuclei.

### 2.9 RNA isolation and quantitative real‐time reverse transcription PCR (qRT‐PCR) analysis

RNA extracted from cell samples was quantified by using a Nanodrop 2000 Spectrophotometer (Thermo Scientific) and reverse transcribed into cDNA using Prime Script RT Master Mix. SYBR Premix Ex Taq (Shiga) was used to perform the quantitative real‐time PCR (qPCR) reaction in triplicate, following the manufacturer's protocol. The following primers were used: *Tnfa* (TNF‐α), forward: TCGTAGCAAACCACCAAGCA, reverse: CCCTTGAAGAGAACCTGGGAGTA; *Il6* (IL‐6), forward: TCCTACCCCAACTTCC AATGCTC, reverse: TTGGATGGTCTTGGTCCTTAGCC; *Il1*b (IL‐1β), forward: CACCTCTCAAGCAGAGCACAG, reverse: GGGTTCCATGGTGAAGTCAAC; and glyceraldehyde 3‐phosphate dehydrogenase (*Gapdh*), forward: GCGAAAGCATTTGCCAAGAA, reverse: GGCATCGTTTATGGTCGGAAC. mRNA levels were normalized to that of GAPDH and presented as relative values using the 2^−ΔΔCT^ method.

### 2.10 Western blotting analysis

Protein samples were extracted with the help of sodium dodecyl sulphate/polyacrylamide gel electrophoresis and transferred to polyvinylidene fluoride membranes. The membranes were probed using primary antibodies recognizing β‐actin (Sigma), zona occludens 1 (ZO‐1), Claudin‐1, Occludin, TLR4, MyD88 and NF‐κB (p65) (all from Cell Signaling Technology).

### 2.11 Statistical analysis

We presented all the data as the mean ± the standard error of the mean (SEM). Statistical analyses were conducted using one‐way analysis of variance (ANOVA) and a significant difference post hoc test. Statistical significance was accepted at *P* < .05.

## 3 RESULTS

### 3.1 The effect of *Spirulina platensis* on bodyweight, insulin intolerance and serum lipid parameters in HFD‐fed rats

**Figure 1:**
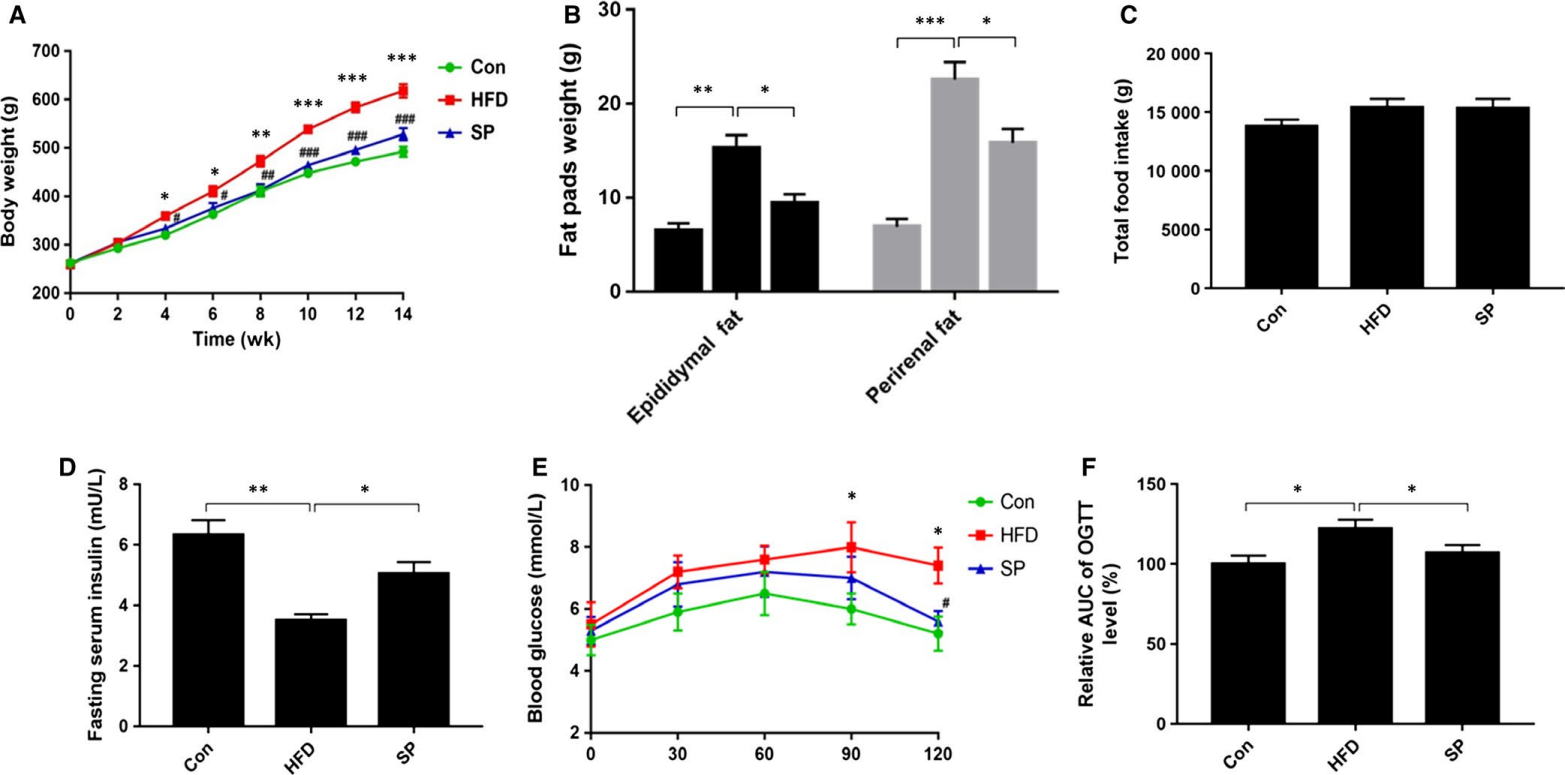
Effect of *Spirulina platensis* on body weight, visceral fat pad and insulin intolerance in rats fed HFD. Con, normal diet‐fed rats; HFD, high‐fat diet‐fed rats; SP, rats fed with HFD supplemented with 3% *Spirulina platensis* (3 g/100 g diet). A, Body weight curve, B, Epididymal and perirenal fat pads weight, C, Total food intake, D, Fasting serum insulin, E, Oral‐glucose‐tolerance test for blood glucose, F, Blood glucose AUC. Values are the mean ± SEM, n ≥ 6, **P < .01, **P < .001, ***P < .001 HFD vs Con; ^#^ *P* < .05, ^##^ *P* < .01, ^###^ *P* < .001, HFD + SP vs HFD

All the rats in the three groups shared the same bodyweight at the beginning of the experiment. However, their bodyweights changed after being fed for 12 weeks. Notably, compared with the control rats, the HFD‐fed rats showed increased bodyweights; however, the weight of the HFD rats fed on *S platensis* decreased (Figure 1A). Additionally, *S platensis* treatment markedly decreased the visceral fat pads weight of the HFD‐fed rats (Figure 1B), without affecting the total amount of food consumed (Figure 1C).

As shown in Figure 1D‐F, *S platensis* treatment efficiently reversed the decrease of serum insulin levels induced by the HFD (Figure 1D). The blood glucose level of OGTT (Figure 1E) and the AUC level of OGTT (Figure 1F) showed that *S platensis* successfully improved insulin sensitivity in HFD‐fed rats.

In terms of the serum lipid parameters, compared with the control group, the rats fed a HFD had significantly increased levels of TG, TC, LDL‐C and FFA; however, these levels decreased after receiving *S platensis* treatment (Table 1).

**Table 1:** Effect of *Spirulina platensis* on serum lipid parameters in rats fed HFD

	Con	HFD	
		–	SP
TG (mmol/L)	0.90 ± 0.05	2.09 ± 0.31***	1.42 ± 0.24^##^
TC (mmol/L)	1.35 ± 0.20	2.29 ± 0.42***	1.70 ± 0.15^###^
HDL‐C (mmol/L)	1.12 ± 0.05	0.75 ± 0.05**	0.99 ± 0.08^#^
LDL‐C (mmol/L)	0.52 ± 0.05	0.76 ± 0.03***	0.55 ± 0.04^##^
FFA (mmol/L)	0.74 ± 0.03	1.38 ± 0.06**	0.92 ± 0.05^##^

Note

Values are the mean ± SEM (n = 8).

Abbreviations: Con, normal diet‐fed rats; HFD, high‐fat diet‐fed rats; SP, rats fed with HFD supplemented with 3% *Spirulina platensi*s (3 g/100 g diet).

** *P* < .01;

*** *P* < .001 HFD vs Con;

^#^ *P* < .05;

^##^ *P* < .01;

^###^ *P* < .001, HFD + SP vs HFD.

### 3.2 The effect *Spirulina platensis* on serum endotoxins and inflammatory cytokines in HFD‐fed rats

**Figure 2:**
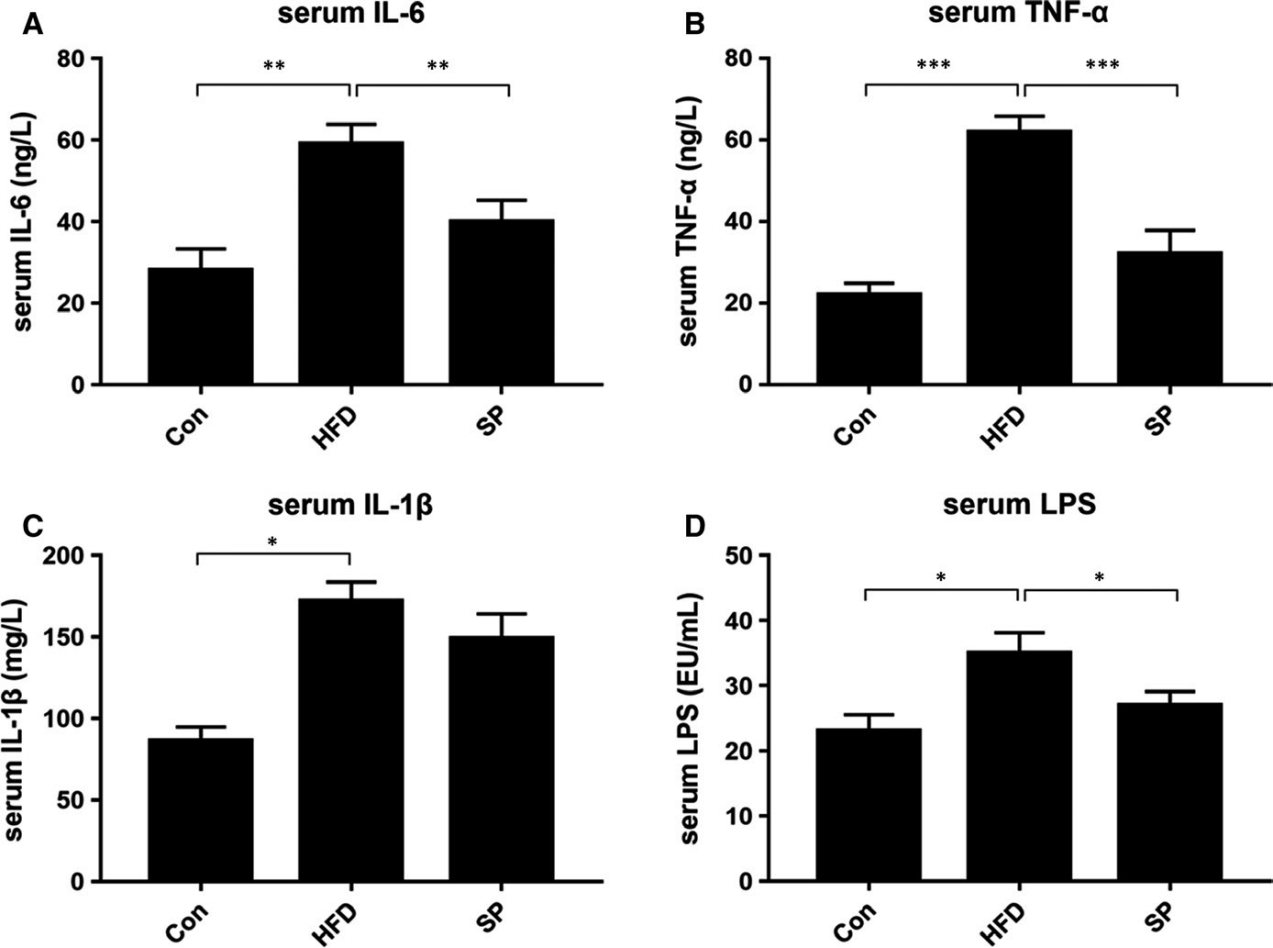
Effect of *Spirulina platensis* on serum inflammatory cytokines and endotoxins in rats fed HFD. Con, normal diet‐fed rats; HFD, high‐fat diet‐fed rats; SP, rats fed with HFD supplemented with 3% *Spirulina platensis* (3 g/100 g diet). The serum levels of (A) IL‐6, (B) TNF‐α, (C) IL‐1β, (D) LPS. Values are the mean ± SEM, n ≥ 6, *P < .05, **P < .01, ***P < .001

Compared with those in the control group, the rats in the HFD group had higher levels of serum IL‐6, TNF‐α and IL‐1β. The increased IL‐6 and TNF‐α levels were reversed by *S platensis* treatment (Figure 2A‐C). Furthermore, the concentrations of serum LPS in the rats fed an HFD were almost twofold higher compared with those fed a non‐HFD diet. The increased LPS concentration was reversed in the rats receiving *S platensis* treatment (Figure 2D).

### 3.3 The effect of *Spirulina platensis* on colon tissue damage in HFD‐fed rats

**Figure 3:**
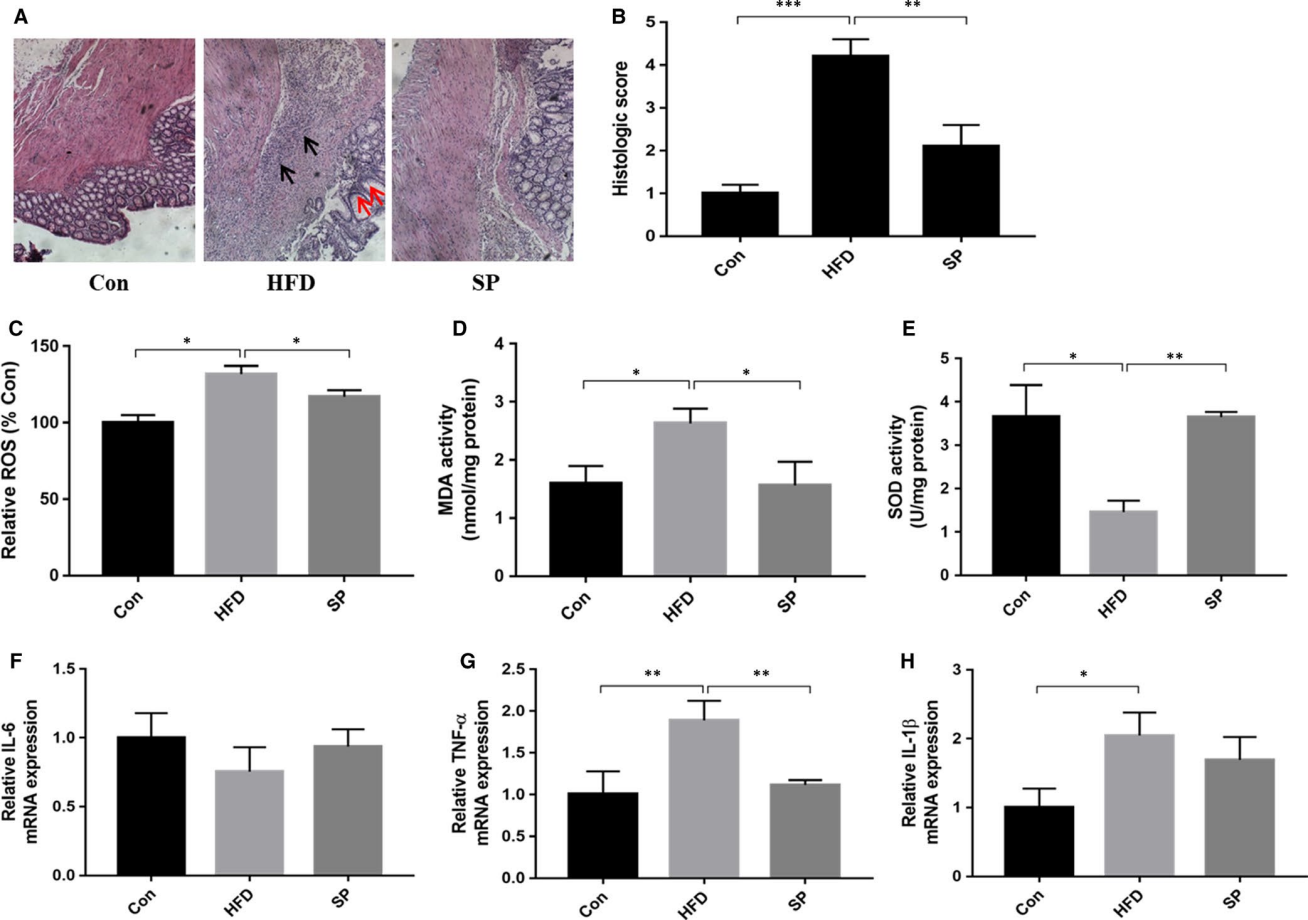
Effect of *Spirulina platensis* on colon tissue damage in rats fed HFD. Con, normal diet‐fed rats; HFD, high‐fat diet‐fed rats; SP, rats fed with HFD supplemented with 3% *Spirulina platensis* (3 g/100 g diet). A, Representative hematoxylin and eosin‐stained sections of colon tissue. B, Statistical analysis of histolopathological score. The activities of (C) ROS and (D) MDA, and (E) SOD capacity. The mRNA levels of (F) IL‐6, (G) TNF‐α, (H) IL‐1β in colon tissue. Values are the mean ± SEM, n ≥ 6, *P < .05, **P < .01, ***P < .001

H&E staining was used to examine the histology and morphology of the colons from the rat groups. In the control group, the colon tissue showed a normal tissue structure, with an intact mucosal epithelium with no obvious inflammatory cell infiltration. However, the colons of the HFD‐fed rats showed marked mucosal inflammatory cell infiltration (black arrow), disordered intestinal gland arrangements, muscular layer thinning (red arrow) and oedema. Based on these results, the colons of rats fed an HFD had a significantly higher histological score compared with that of the control group (Figure 3A,B). Additionally, *S platensis* treatment decreased the level of inflammatory cell infiltration and normalized the thickness of the colonic muscular layer in the HFD‐fed rats leading to a lower histological damage score (Figure 3A,B). In the colon tissue, ROS were by 1.3‐fold in the HFD group compared with that in the control group (Figure 3C). Oxidative stress‐induced MDA levels increased significantly in the HFD group compared with that in the control group (Figure 3D); however, the levels of the antioxidant enzyme SOD decreased significantly (Figure 3E). *S platensis* treatment effectively decreased the ROS and MDA levels, and elevated SOD activity compared with those in the HFD group (Figure 3C‐E). Furthermore, *S platensis* significantly ameliorated the increased mRNA levels of *Tnfa* (Figure 3G) and *Il1b* (Figure 3H) compared with those in the HFD group, but did not affect *Il6* (Figure 3E). *S platensis* treatment also largely attenuated the mRNA level of *Tnfa* compared with the rats fed an HFD (Figure 3G). The results indicated that *S platensis* could effectively alleviate the oxidative and inflammatory damage to the colon induced by the HFD.

### 3.4 *Spirulina platensis* modulated the gut microbiota in HFD‐fed rats

**Figure 4:**
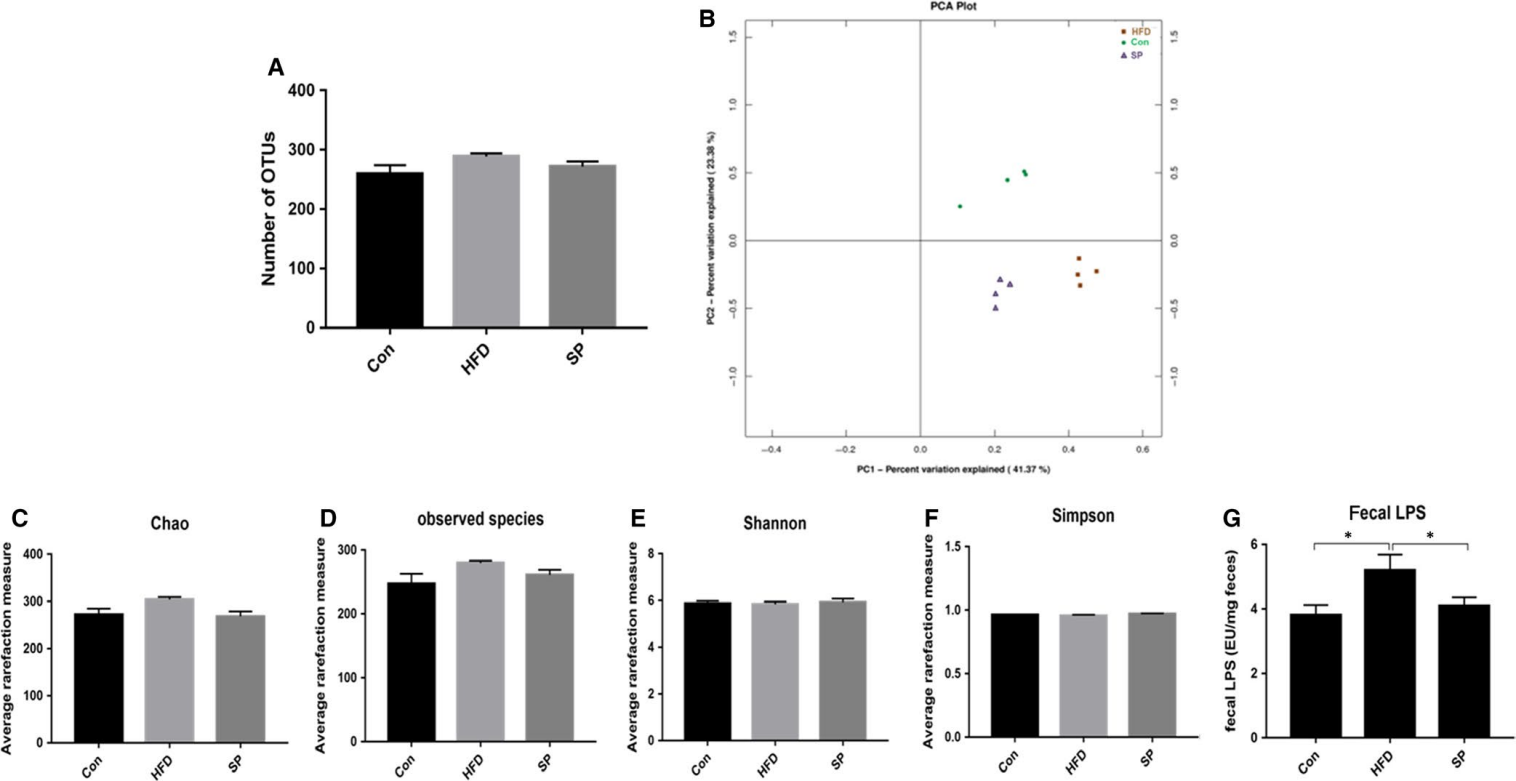
Effect of *Spirulina platensis* on gut microbiota modulation in rats fed HFD. Con, normal diet‐fed rats; HFD, high‐fat diet‐fed rats; SP, rats fed with HFD supplemented with 3% *Spirulina platensis* (3 g/100 g diet). A, Number of OTUs. B, UniFrac‐based PCoA plots. Alpha diversity (C) Chao index. D, Observed species index. E, Shannon index. F, Simpson index. G, LPS. Values are the mean ± SEM, n = 4

Sequencing of the 16S rRNA gene resulted in a total of 783 410 clean (approximately 97 926 ± 456 per sample) for downstream analysis. The sequences could be clustered into 215‐298 OTUs per sample at the 98% similarity level (365 OTUs were generated from all the samples). Among the groups, the number of OTUs (Figure 4A) was not significantly different (*P* > .05). PCA divided the data from the three groups and classified them into three different clusters (Figure 4B). The alpha diversity analysis results are shown in Figure 4C‐F *S. platensis* had no effect on the bacterial biodiversity of colonic contents. Consistent with the blood samples, the concentrations of faecal LPS in the rats fed an HFD were almost 1.5‐fold higher compared with those fed a non‐HFD diet. The increased LPS concentration was reversed in the rats receiving *S platensis* treatment (Figure 4G).

**Figure 5:**
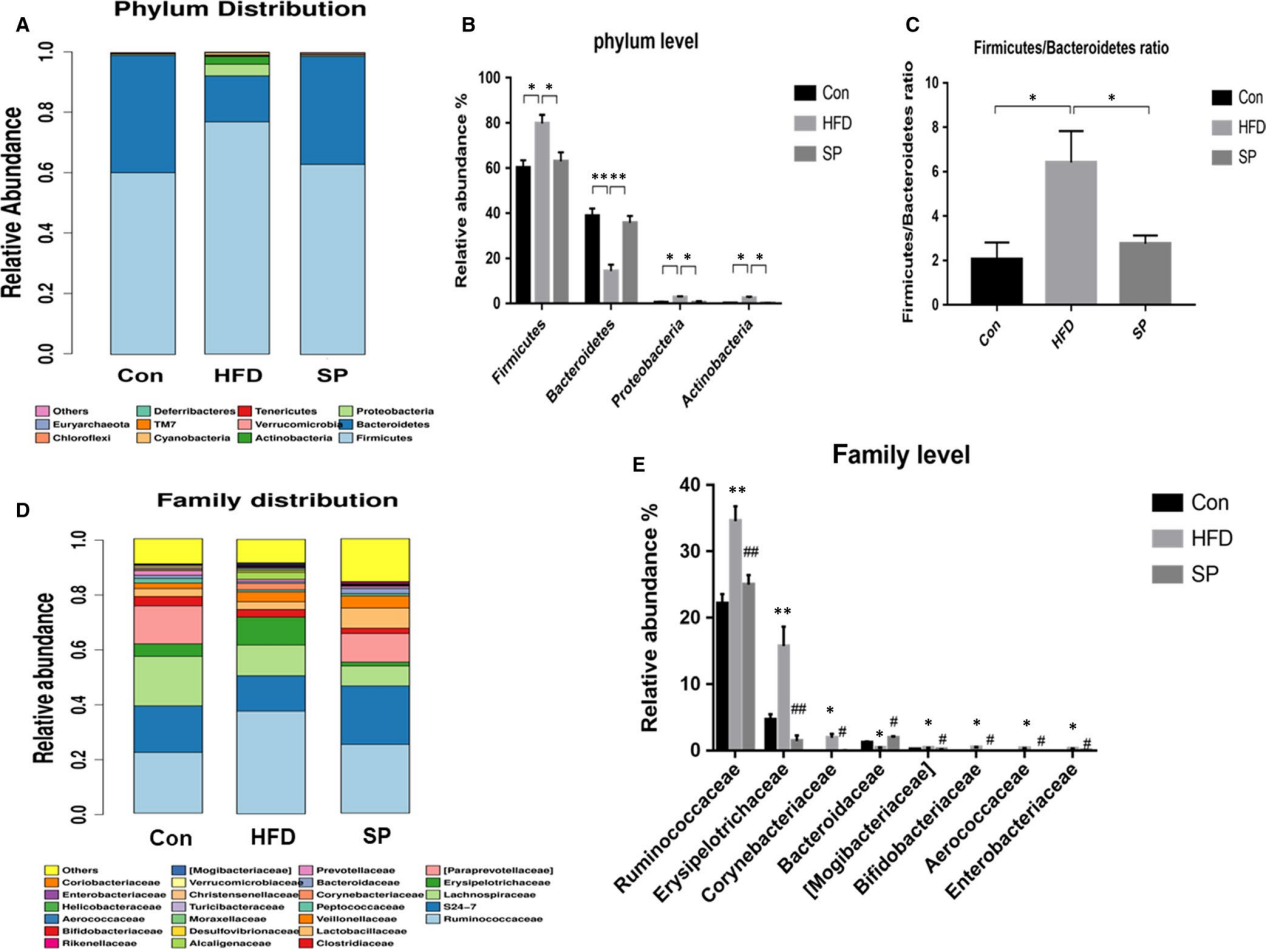
Effect of *Spirulina platensis* on gut microbiota modulation in rats fed HFD. Con, normal diet‐fed rats; HFD, high‐fat diet‐fed rats; SP, rats fed with HFD supplemented with 3% *Spirulina platensis* (3 g/100 g diet). A, Bacterial taxonomic profiling in the phylum level of intestinal bacteria. B, Statistic analysis of bacterium in the phylum level. C, The Firmicutes‐to‐Bacteroidetes ratios. D, Bacterial taxonomic profiling in the family level of intestinal bacteria. E, Statistic analysis of bacterium in the family level. Values are the mean ± SEM, n = 4. *P < .05, **P < .01, ^#^ *P* < .05 compared with the HFD group

In the rat colon samples, 11 major phylla dominated the faecal microbiota: *Firmicutes*, *Bacteroidetes*, *Proteobacteria*, *Actinobacteria*, *Verrucomicrobia*, *Tenericutes*, *Cyanobacteria*, *TM7*, *Deferribacteres*, *Chloroflexi* and *Euryarchaeota* (Figure 5A). The abundance of *Firmicutes, Proteobacteria* and *Actinobacteria* was enriched after HFD treatment (Figure 4B), whereas the relative abundance of *Bacteroidetes* decreased significantly (Figure 5B). *S platensis* altered the colonic bacterial communities. The rats fed *S platensis* had a significantly lower levels of the *Firmicutes, Proteobacteria* and *Actinobacteria* compared with those in the control (Figure 5B). *S platensis* increased the relative amount off the *Bacteroidetes* compared with that in the HFD group (Figure 5B). The HFD group experienced a significantly higher ratio of *Firmicutes*/*Bacteroidetes* compared with that in the control group, which decreased significantly after *S platensis* treatment compared with the HFD‐fed rats (Figure 5C). Twenty‐five species of bacteria were detected at the family level, among them the levels of eight species were altered in the *S platensis* compared with that in the HFD group (Figure 5D). In the control group, *S platensis* treatments altered all the microbiota relative abundance in the same direction (Figure 5E), suggesting that treatment with *S platensis* could effectively modulate the gut microbiota. In the *Firmicutes*, the levels of three species were reversed by *S platensis*: *Ruminococcaceae*, *Erysipelotrichaceae* and *Aerococcaceae*. In the *Bacteroidetes*, the HFD‐induced change in the relative abundance of *Bacteroidaceae* was reversed by *S platensis* treatment. *S platensis* treatment reversed the changes in three genera, namely *Corynebacteriaceae* and *Bifidobacteriaceae* in the *Actinobacteria* phylum, and *Enterobacteriaceae* in the *Proteobacteria* phylum, compared with the relative abundance in the HFD group (Figure 5E). Thus, treatment with *S platensis* restored the overall composition of the gut microbial community in HFD‐fed rats to that of the control group.

### 3.5 The effect of *Spirulina platensis* on colonic tight junction‐related parameters and TLR4‐MyD88‐NF‐κB pathway in HFD‐fed rats

**Figure 6:**
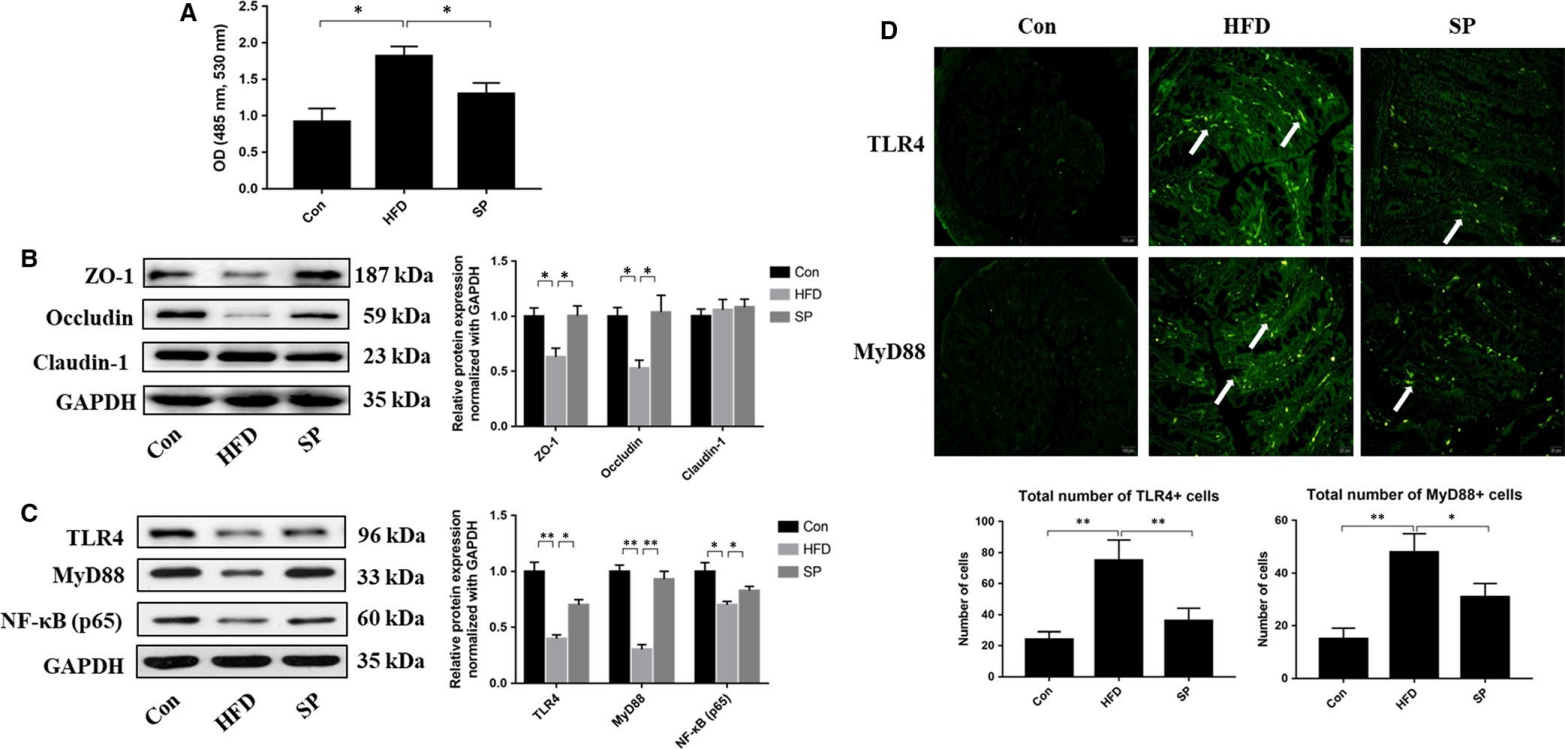
Effect of *Spirulina platensis* on the level of colonic tight junction protein in rats fed HFD. Con, normal dietfed rats; HFD, high‐fat diet‐fed rats; SP, rats fed with HFD supplemented with 3% *Spirulina platensis* (3 g/100 g diet). A, Gut permeability measured by appearance of FITC‐labeled dextran in plasma. B, Representative images of the western blotting and statistical analysis for zonula occluden‐1 (ZO‐1), Occludin and Claudin‐1 using GAPDH as the loading control. C, Representative images of the western blotting and statistical analysis for TLR4, MyD88 and NF‐κB (p65) using GAPDH as the loading control. D, Representative immunofluorescence staining and statistical analysis for TLR4 and MyD88. Nuclei were identified by DAPI. Scale bar = 30 μm. Values are the mean ± SEM, n ≥ 6, *P < .05, **P < .01

In vivo assessment of gut permeability revealed a significant increase in serum FITC‐dextran in HFD‐fed rats, while *S platensis* treatment decreased the impairment of intestinal barrier functions measured by lower serum FITC‐dextran than the HFD group (Figure 6A). We then analysed the expression level of tight junction proteins to assess gut permeability. The rats receiving an HFD showed significantly lower protein levels of ZO‐1 and Occludin compared with those in the control rats. ZO‐1 and Occludin levels increased in the *S platensis*‐treated rats (Figure 6B). Western blot results revealed an increase of TLR4, MyD88 and NF‐κB (p65) protein expression in rats fed an HFD *S platensis* treatment effectively decreased the TLR4, MyD88 and NF‐κB (p65) protein expression compared with those in the HFD group (Figure 6C). Furthermore, immunohistochemical assessment of colonic TLR4 and MyD88 expression showed that an HFD significantly increased the positive area of TLR4 and MyD88 compared with that in the control group. *S platensis* treatment decreased the abundance of TLR4 and MyD88 compared with that in rats fed an HFD (Figure 6D). This result further indicated that *S platensis* improved the TLR4‐MyD88‐NF‐κB pathway related‐gut hyperpermeability associated with an HFD. Therefore, chronic metabolic inflammation induced by an HFD is perhaps driven by heightened gut permeability. The positive effect of *S platensis* demonstrated in the present study is probably achieved via relief of this damage.

## 4 DISCUSSION

The present study had four key findings. First, *S platensis* can help the prevent obesity resulting from a HFD. Second, it reduced chronic low‐grade inflammatory responses. Third, it lowered the expression of colon tight junction proteins. Lastly, it contributed to gut microbiota modulation. Specifically, the gastrointestinal tract has the largest potential to produce circulating inflammatory cytokines. Lim et al^30^ showed that an HFD created a negative effect on gut hyperpermeability and endotoxaemia‐induced inflammation. Moreover, dietary food can shape the microbiota and promote its modification, thereby increasing the amount of disease‐inducing microbes compared with benign microbes.^31^, ^32^ In addition, after an HFD induces harmful bacteria, it will further negatively affect the intestinal epithelial cells or trigger the production of harmful metabolites, causing gut hyperpermeability, which results in LPS and pro‐inflammatory cytokines leakage from the colon into circulation. There is a network of factors that, in addition to the action of LPS, contributes to the development of insulin resistance, such as elevated plasma levels of FFA and mitochondrial dysfunction and hormone levels (reduced adiponectin or leptin resistance).^33^

Accumulating evidence shows that restricting dietary intake can modulate the gut microbiota and inhibit obesity and its associated diseases.^34^ Cani et al^29^ studied diet‐induced mice with obesity and demonstrated that antibiotics improved the chronic metabolic low‐grade inflammatory responses. The regulatory efficacy of the gut microbiota can also be demonstrated via nutritional interventions, which reduce the symptoms of metabolic disorders caused by HFDs. Previous studies reported that *S platensis* could ameliorate the metabolic inflammation induced by an HFD to protect animals^35^ and boost probiotic population, such as *Bifidobacterium* and *Lactobacillus*, in vitro.^20^ In consistent with previous studies, we shown that *S platensis* treatment significantly decreased the bodyweight, fat pads weight, serum metabolic makers levels, improve insulin sensitivity and chronic inflammatory states of HFD‐treated rats. These results demonstrated that *S platensis* could be used as an effective agent in ameliorating the HFD‐induced effects.

The gut microbiota of animals receiving an HFD comprised an increased intestinal ratio of *Firmicutes* to *Bacteroidetes*, indicating that these major altered phyla are important in metabolic disorders caused by HFDs.^36^ Nutritional interventions contributed to reversing the ratio and ameliorating disorders caused by obesity in animals on an HFD. Specifically, the most common nutritional interventions that show this effect are resveratrol,^37^ pomegranate peel polyphenols^38^ and dietary fibre.^39^ According to our results, *S platensis* reversed the increased ratio of *Firmicutes* to *Bacteroidetes* induced by the HFD. At the family and phylum levels, *S platensis* demonstrated its efficacy in restoring the gut microbiota imbalance induced by the HFD. At family level, the HFD altered the abundance of two main Gram‐negative bacteria, the *Enterobacteriaceae* and *Bacteroidaceae*. The relative abundance of the *Enterobacteriaceae* increased, while that of the *Bacteroidaceae* decreased. The *Enterobacteriaceae* function as a strong agonist of TLR4.^40^ In addition, the LPS lipid A structures from the *Enterobacteriaceae* exhibit a higher binding with members of the TLR4 activation pathway than do the lipid A structures of the *Bacteroidaceae*. Thus, dysbiosis‐related shifts in the intestinal microbiome that increase the proportions of the *Enterobacteriaceae* correlate with more potent lipid A structures, resulting in the intestinal tissue becoming more susceptible to TLR4 activation by decreasing the amount of LPS required to activate TLR4 and producing a strong response to inflammation. In a previous study, *Ruminococcaceae* and *Erysipelotrichaceae* were enriched in the faeces of mice fed with an HFD and in db/db mice compared with the control mice ones and were associated positively with obesity phenotypes.^41^ The results of the present study agree with these previous findings: In rats fed an HFD, the diversity and amount of *Ruminococcaceae* and *Erysipelotrichaceae* were increased compared with those in the control group, while *S platensis* treatment had the opposite effect. This indicated that *S platensis* treatment modulated the imbalance of gut microbiota imbalance caused by an HFD and might be explanation for *S platensis’* reduction of HFD‐induced intestinal tissue damage. Those results were consistent with previous studies that *S platensis* and its components have benefit effect on HDF‐induced gut microbiota dysbiosis.^20^, ^21^, ^22^ However, the regulation of intestinal barrier function as well as the improvement in intestinal tissue damage under HFD by spirulina platensis and its mechanism has not yet been studied.

There are two important features of the intestinal problems caused by HFD‐induced obesity: Oxidative stress and inflammatory activation.^42^ Studies have proved that oxidative damage can increase intestinal permeability, for example, in Caco‐2 cells, hydrogen peroxide–induced inflammatory cytokines interferon gamma (IFN‐γ) and TNF‐α contributed to intestinal hyperpermeability.^43^ Additionally, stress‐induced inflammation and intestinal oxidative damage affects the expression of junction proteins.^44^ Based on this, we hypothesized that in addition to affecting the gut microbiota, *S platensis* could also alleviate HFD‐induced oxidative damage and intestinal tissue inflammation. In the present study, *S platensis* restored the decreased levels of tight junction proteins. In addition, *S platensis* significantly inhibited the expression levels of TLR4, MyD88 and NF‐κB in the colon compared with those in rats fed an HFD. This suggested that the *S platensis* suppressed the inflammatory response and the increase in intestinal pro‐inflammatory cytokines via blocking the TLR4/MyD88/NF‐κB signalling pathway, which is consistent with the decreased serum levels of TNF‐α, and IL‐6 in the *S platensis* fed rats compared with the control group.

We also believe that treatment with *S platensis* would be safe because throughout the experimental period, no toxic effects of *S platensis* were noted, regardless of the animals’ weight, food intake, serum biochemistry or capability of completing daily activities. However, we suggest that a more throughout assessment of its toxicity be carried out before adding it to the human diet, because currently, we lack clinical research and evidence to evaluate its side effects when used to treat humans.

**Figure 7:**
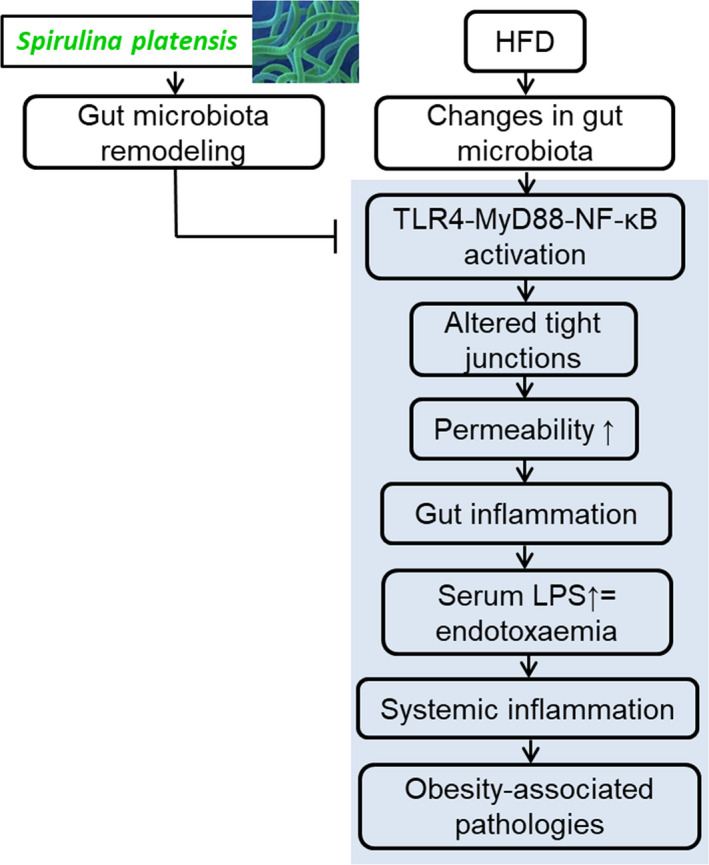
Proposed mechanism by which *Spirulina platensis* alleviates chronic low‐grade inflammatory responses with modulation of gut microbiota and intestinal permeability in rats fed a HFD

In summary, we concluded that the nutritional value of *S platensis* lies in its potent efficacy to treat obesity‐induced systematic inflammation driven by an HFD, particularly through the mechanism of reducing intestinal damage and correcting the gut microbiota imbalance (Figure 7). Therefore, we suggest that the prebiotic effects of *S platensis* should be leveraged to improve gut health and prevent intestinal dysfunction. These results provide the basis for the further manufacture of *S platensis* as well as the study the mechanism of pomegranate extracts.

## CONFLICT OF INTEREST

The authors declare no competing conflict of interest.

## AUTHOR CONTRIBUTION

**Ting Yu:** Conceptualization (lead); Formal analysis (lead); Investigation (lead); Writing‐original draft (lead). **Yan Wang:** Data curation (equal); Investigation (equal). **Xiaosu Chen:** Data curation (equal); Investigation (equal). **Wenjie Xiong:** Methodology (equal); Resources (equal). **Lin Lin:** Conceptualization (equal); Funding acquisition (lead); Writing‐review & editing (equal). **Yurong Tang:** Conceptualization (equal); Funding acquisition (equal); Methodology (equal); Writing‐review & editing (lead). 

## Data Availability

All data generated or analysed during this study are included in this article.
